# Exosome-Transmitted *miR-128* Targets CCL18 to Inhibit the Proliferation and Metastasis of Urothelial Carcinoma

**DOI:** 10.3389/fmolb.2021.760748

**Published:** 2022-01-04

**Authors:** Donghao Shang, Yuting Liu, Zhenghao Chen

**Affiliations:** ^1^ Department of Urology, Beijing Friendship Hospital, Capital Medical University, Beijing, China; ^2^ Department of Pathology, Capital Medical University, Beijing, China

**Keywords:** exosome, urothelial carcinoma, miR-128, chemokine (C-C motif) ligand 18, proliferation, metastasis

## Abstract

**Objective:** To investigate the regulatory function of exosome-transmitted *miR-128* and chemokine (C-C motif) ligand 18 (CCL18) on urothelial carcinomas (UCs).

**Methods:** Tumor tissues, paracancerous tissues, and serum were collected from 20 patients with UCs (diagnosed at Beijing Friendship Hospital, Capital Medical University). CCL18 was detected by immunohistochemistry and ELISA. PCR was used to measure the expression levels of CCL18 and *mir-183*, *miR-128*, *mir-33a* in UCs. We acquired exosomes from mesenchymal stem cells and synthesized exosomes overexpressing *miR-128* (HMSC-128-EV). The effects of *miR-128* on the migration and invasion abilities, apoptosis and epithelial-mesenchymal transition of BUC T24 cells were investigated by co-culturing HMSC-128-EV. The therapeutic potential of *miR-128* on disease models was explored by injecting HMSC-128-EV into nude mice.

**Results:** The expression of CCL18 in UCs was significantly higher than that in normal tissues (*p* < 0.05), and the serum level of CCL18 in patients with UC was significantly increased compared with those in healthy controls (*p* < 0.05). CCL18 overexpression or downregulation enhanced or suppressed the proliferation, migration and invasion of BUC T24 cells, resectively (*p* < 0.05). The exosome-transmitted miR-128 can inhibit cell proliferation (*p* < 0.05), invasion (*p* < 0.05), and migration (*p* < 0.05) in UCs, and these effects can be reversed by CCL18. In terms of apoptosis, *miR-128* was able to promote the occurrence of BUC T24 apoptosis (*p* < 0.05), which can also be reversed by CCL18. In addition, *miR-128* can inhibit the proliferation (*p* < 0.05) and metastasis (*p* < 0.05) of UCs in nude mice.

**Conclusion:** The *miR-128* inhibits the proliferation, invasion, migration of UCs, and promotes its apoptosis by regulating CCL18 secretion.

## Introduction

Urothelial carcinomas (UCs) are the ninth most common form of manignancies, killing over 165,000 patients annually around the world (Rosenberg et al., 2016). Urinary bladder cancer accounts for 90–95% of all UCs and is the most common malignancy of UCs (Rouprêt et al., 2021). In terms of the histological type, urothelial and squamous carcinoma account for about 90 and 5% of all bladder cancers, respectively (Sjödahl et al., 2012). UCs of the bladder can be classified as nonmuscle-invasive bladder cancer (NMIBC) and muscle-invasive bladder cancer (MIBC) based on pathologic stage. For the NMIBC, the recurrence rate is more than 50%, and disease progression will develop for 10–15% of patients (Têtu, 2009; Hedegaard et al., 2016). Currently, cisplatin-based combination chemotherapy has been used as the standard treatment for unresectable and metastatic/advanced UC, but patients who relapse after first-line treatment or have progression while receiving first-line treatment have a particularly poor prognosis, and second-line chemotherapy also shows only moderate efficacy, with an ORR of 12% and a median OS of 5–7 months (Kim and Seo, 2018). Thus, it is of great practical significance to develop drugs that can inhibit the proliferation and metastasis of UCs of the bladder.

The development and metastasis of tumors are the results of the interaction between tumor cells and the microenvironment. Exosomes are newly discovered extracellular vesicles in the tumor microenviroment, which play important roles in the cell proliferation, apoptosis, metastasis and other biological behaviors of tumor (Kahroba et al., 2019). These vesicles are 30–150 nm in diameter (Maia et al., 2018), and can be detected in various body fluids including blood, interstitial fluid, and urine (Harding et al., 2013). The contents of exosomes are proteins, DNAs, micro-RNAs (miRNAs), long non-coding RNAs, circular RNAs and other molecules (Rajagopal and Harikumar, 2018). Exosome-transmitted miRNAs have been proved to participate in regulating the activity of bladder cancer cells through different signaling pathways (Cai et al., 2020). The *miR-128* is a kind of miRNA enriched in the brain. Previous studies have shown that *miR-128* plays an important role in the development of the nervous system and the maintenance of its normal function (Persengiev et al., 2012). miR-128 can also be found to be abnormally expressed in the serum of some patients with malignant tumors (Roth et al., 2011), and is involved in regulating the occurrence and development of various cancers. The CCL18 is a target gene of *miR-128*, and by regulating the expression of CCL18, *miR-128* can regulate tumor invasion and metastasis (Song et al., 2018; Korbecki et al., 2020). However, the effect and mechanism of exsome-transmitted *miR-128* on the proliferation and metastasis of UCs has not been studied. Further exploration of the therapeutic potential of *miR-128* will provide entirely new options for the treatment of metastatic/advanced UC.

The chemokine (C-C motif) ligand 18 (CCL18) plays an important role in the progression of cancers, and this chemokine is mainly produced by tumor-associated macrophages (TAMs) (Lin et al., 2015; Ma et al., 2019). Previous studies have found that CCL18 is up-regulated in malignant tumors, and can promote the invasion and metastasis of tumor cells, which is a potential pathogenic molecule of urothelial carcinoma (Bo et al., 2018; Liu T. et al., 2019). The target genes of CCL18 were predicted by miRanda and Targetscan online software, and it was found that CCL18 had binding sites with *miR-128*, miR-183 and miR-33a. Only *miR-128* was negatively correlated with the expression of CCL18 in UCs cells, and CCL18 was not correlated with the expression of the other two miRNAS in UCs.

In this paper, we found that CCL18 was highly expressed in UCs, and inhibition of CCL18 could inhibit the biological activity of UCs. Also, as a downstream molecule of *miR-128*, CCL18 participates in the regulation of the biological process of UCs. CCL18 and *miR-128* are of great significance as new targeting molecules for UCs treatment.

## Materials and Methods

### Materials

Tumor tissues, paracancerous tissues, and serum were obtained from 20 patients with UCs diagnosed in Department of Urology, Beijing Friendship Hospital. The inclusion criteria were patients who were diagnosed with UCs and had not received any treatment such as chemotherapy, radiotherapy and biological drugs (monoclonal antibodies) before sampling. The human urothelial carcinoma cell line BUC T24 was purchased from Gai Ning Biological Company. Human bone marrow-derived mesenchymal stem cells (HMSCs) were purchased from Shanghai Yaji Biotechnology Co., Ltd. This study conformed to the Declaration of Helsinki and was reviewed and approved by the Ethics Committee of Beijing Friendship Hospital, Capital Medical University, and all patients signed written informed consent.

### Immunohistochemistry

Paraffin sections were deparaffinized with xylene for 10 min and rehydrated with graded alcohol. Antigen retrieval was performed by boiling the sections in 0.1 M citric acid buffer (pH 6.0) at 120°C for 10 min in a decloaking chamber (Biocare Medical, Walnut Creek, CA). After natural cooling, the sections were washed 3 times with PBS for 5 min each time. Then, the sections were stained with rabbit anti-CCL18 IgG Ab (20 μg/ml, Abcam) and rabbit anti-Ki67 (2 μg/ml, Abcam). Stayed overnight on a shaker at 4°C. After incubation, the slices were washed with PBS for 3 times, 5 min each time, then added hypersensitivity two-step immunohistochemical detection reagent, incubated at room temperature for 20 min; washed 3 times with PBS, 5 min each time, DAB (Sigma-Aldrich) was used for color development. Then hematoxylin complex dyeing, gradient alcohol dehydration, xylene transparent and sealed. The sections were observed, photographed, and counted under a light microscope.

The apoptosis of tumor was detected by TUNEL staining (Abcam), and the operation procedure was referred to the instruction manual.

### Enzyme-Linked Immunosorbent Assay

Serum CCL18 levels were determined using Duoset ELISA assays (RandD Systems, Minneapolis, MN, United States) consulting the manufacturers instructions. The serum sample was centrifuged and the supernatant was added into the well plate. Each well was sealed with blocking solution for 2 h, washed with PBST solution, and incubated with antibodies for 2 h. Finally, the substrate TMB-hydrogen peroxide urea solution was added, and the color was developed at room temperature for 10 min. The OD_450_ value was detected by a microplate reader.

### Reverse Transcription-Polymerase Chain Reaction

BUC T24 cells were incubated until cell confluency reached 50%, and then transfected separately with each group using Lipofectamine 2000 (ThermoFisher). Group A was the control group. Cells of group B was transfected with 2 μg/μL PCDNA3.1-CCL18 (GenePharma). Cells of group C was transfected with 50 nM siRNA (GenePharma) for knockdown of CCL18. After 24 h, the cells were collected to perform RT-PCR. According to the manufacturer’s instruction, total RNA was reversely transcribed into cDNA by using the ReverTra Ace qPCR-RT Kit (Toyobo, Osaka, Japan) in line. PCR was carried out on CFX96 Touch Real-Time PCR Detection System (Bio-Rad, Hercules, CA, United States) by using SYBR-Green RealMastcrMix (Bio-Rad) to conduct amplified detection.

CCL18 primer sequences:

Forward: 5′-AAA​CTC​GAG​CTG​CCC​AGC​ATC​ATG​AAG​G-3′,

Reverse: 5′-TTT​GGA​TCC​CCT​CAG​GCA​TTC​AGC​TTC​AG-3′.

*miR-183* primer sequences:

Forward: 5′- CGT​TGG​ATT​CCT​ATG​GCA​CTG​GT-3′,

Reverse: 5′- TTC​AAG​CAG​GGT​CCG​AGG​TAT​TC-3′.

*miR-128* primer sequences:

Forward: 5′- TCACAGTGAACCGGTCTCTTT-3′,

Reverse: 5′- GCTGTCAACGATACGCTACG-3′.

*miR-33a* primer sequences:

Forward: 5′- GGTGCATTGTAGTTGCATTGC-3′,

Reverse: 5′- GTGCAGGGTCCG AGGTATTC-3′.

### Western Blotting

Cell lysates were collected by digesting with RIPA buffer (Beyotime, Nanjing, China) and protease inhibitors (Sigma-Aldrich, United States). BCA kit determined protein concentration (Thermo Fisher, United States). Thirty μg protein were loaded on 10% or 15% SDS-PAGE gels and transferred onto nitrocellulose membranes. The membranes were incubated with specific first antibodies and corresponding second antibody. Primary antibodies purchased from Abcam (Cambridge, United Kingdom) included rabbit anti-CCL18 (2 μg/ml), rabbit anti-Bim (1:500); rabbit anti-β-catenin, rabbit anti-Ecadherin, rabbit anti-N-cadeherin, rabbit anti-Bax (1:1,000); rabbit anti-Vimentin, rabbit anti-Bad, rabbit anti-Bcl2, rabbit anti-caspase 3, rabbit anti-caspase 9 (1:2,000). Secondary antibodies included goat anti-mouse IgG (ab6789, 1:5,000; Abcam) and goat ant-rabbit IgG (ab6721, 1:5,000; Abcam).

### Cell Viability Detection

Cell viability was measured using Cell Counting kit-8 (RandD). A single cell suspension (5 × 10^3^/ml, 100 μL) was seeded into a 96-well plate. Subsequently, 10 μL CCK-8 reagent was added to each well and the plates were incubated for 2 h at 37 °C. Finally, the absorbance was measured at 450 nm using a scanning microplate reader (ThermoFisher).

### Transwell Cell Migration and Invasion Assay

Transwell holes were pretreated with 1:8 diluted Matrigel (BD), then the above cells were inoculated in 24-well plates with 2.5 × 10^5^/well, culturing for 8 h. The number of migration cells was detected by crystal violet staining. For the invasion assay, Matrigel diluted with 100 μL was added in the bottom center of the upper Transwell invasive chambers, incubating for 4 h at 37°C to form a gel. Then, the cells were added and treated in the same steps as the migration experiment. After that, the cells on the lower surface were fixed with 100% methanol for 30 min and then the cells were stained with 0.05% crystal violet for 30 min. Images of the invasion and migration cells were taken under a microscope. All of the experiments were performed in triplicate.

### Prediction of miRNA Targeting 3′UTR of Human CCL18 Gene

The miRanda and Targetscan online softwares were used for prediction of target genes.

Firstly, log in to miRanda (http:/www. Microrna.org/ microrna/home.do). Search for miRNAs whose target gene was human CCL18mRNA (NM002988). The evaluation indexes of the interaction between miRNA and target gene mRNA included MIRSVR score and Phastcons score. Then login to Targetscan (http://www.targetscan.org/vert_80/) online searching for miRNAs whose target gene is human CCLI8 (ENST0000921.3). The evaluation index of the combination of miRNA and target gene mRNA included Total context++ score or Aggregat PCT.

### Preparation and Identification of Exosomes Overexpressing *miR-128* (HMSC-128-EV)

HMSCs were plated into six well culture-plates with 1 × 10^6^/well for 24 h, and then the culture medium was replaced using the mixture including fresh medium and adenovirus with *miR-128* overexpression for 24 h. The supernatant was collected and used to extract exosomes overexpressing *miR-128* (HMSC-128-EV) according to Invitrogen Kit (Invitrogen 4478359 Total Exosome Isolation Reagent) for total exosome isolation reagent. To confirm that the extracts were indeed exosomes, extracts were examined for CD9 and CD63 expression using Western blotting and flow cytometry, electron microscopy was used to visualize extract morphology, dynamic light scattering (DLS) was used to measure the size of the extracts, and laser light scattering was used to measure the zeta potential of the extracts. Further, exosome RNA was extracted using the Exosome DNA Extraction Kit (Biovision) and detected the expression of *miR-128* by the RT-PCR.

### Co-culture of UCs and Exosomes

DiL dye was added to HMSC-EVs and HMSC-128-EV. Exosomes were washed with PBS after staining for 30 min, and the pellet was taken after centrifugation at 10,000 g for 60 min. After resuspending exosomes, they were cocultured at a concentration of 20 μg/ml with BUC T24 for 0.5, 1, and 4 h, respectively. Finally, the uptake of exosomes by cells was observed by confocal microscopy and flow cytometry.

After determining that exosomes could be absorbed by UCs, BUC T24 were divided into four groups (Table 1). After 24 h of treatment, the expression of *miR-128* was detected by RT-PCR, and the content of CCL18 in the supernatant was detected by ELISA.

**TABLE 1:** Co-culture of exosomes grouping.

Group	Tab
BUC T24	Control
BUC T24 treated with HMSC-EVs	HMSC-EV
BUC T24 treated with HMSC-128-EVs	HMSC-128-EV
BUC T24 treated with HMSC-128-EVs and CCL18	HMSC-128-EV + CCL18

### Colony Formation Assay

The grouping of cells was the same with Table 1. Cells were trypsinized and washed twice with phosphate-buffered saline. The number of cells was counted following staining with trypan blue at room temperature and the cells were prepared into a suspension with a density of 0.5 × 10^3^ cells/ml. The cell suspension (2 ml) was inoculated into 6-well plates, followed by incubation under normal conditions at 37°C. Half the medium was replenished on day 5. The medium was discarded on day 14 and cells were washed once with phosphate-buffered saline. Cells were fixed with methanol at room temperature for 10 min and stained with crystal violet at room temperature for 5 min. Following extensive washing with phosphate-buffered saline and the cells were observed under a microscope (Leica). Five fields were selected for colony counting. The colony formation rate was then calculated using the following equation: colony formation rate = (number of clones)/(number of seeded cells) ×100%.

### Wound Healing Assay

The grouping of cells was the same with Table 1. The cells inoculated in 6-well plates were culture to achieve 60% cell fusion, discarding the culture medium and washed in PBS for 3 times. The sterilized micropipette tips were used to quickly draw a straight line at the bottom of the culture dish, washed with PBS for 3 times. After incubation for 0, 6, 12, and 24 h, respectively, the images were collected.

### Flow Cytometry

The grouping of cells was same with Table 1. After treatment for 24 and 48 h, Annexin/PI staining was used to detect cell apoptosis by flow cytometry.

### Tail-Vein Injection of Exosomes in Nude Mice

The BUC T24 cells were inoculated subcutaneously in nude mice. Then, 15 nude mice with similar tumor sizes were selected and randomly divided into three groups with five in each group. PBS (Control), HMSC-EV and HMSC-128-EV were injected intravenously, respectively, every 2 days (200 μg per mice). The weight of nude mice and tumor volume were measured to draw the weight curve of mice and the tumor growth curve. On the 16th day, the tumors were taken out and weighed, performed with TUNEL and Ki67 staining.

### Fluorescent Imaging of Nude Mice

The experimental grouping was the same as mentioned above. Female nude mice, aged 6–8 weeks, were injected with luciferase labeled BUC T24-Luc cells via tail-vein with 2 × 10^7^ cells in 200 μL PBS. Nude mice were treated in the way described above and scanned with fluorescence imaging weekly. Before imaging, the mice were anesthetized by intraperitoneal injection of 0.7% pentobarbital sodium with 10 μL/g and then injected with luciferase substrate (150 mg/kg). After 10 min, the mice were placed in a fluorescence imager for fluorescence observation and quantitative analysis. Fluorescence images were obtained and average fluorescent value in select regions of interest (ROI) were quantified with IVIS® software. On the 28th day, the nude mice were killed and their lungs were taken out for imaging.

### Statistics

SPSS 17.0 for Windows (IBM Corporation, Armonk, NY) and GraphPad Prism (GraphPad Software, Inc., San Diego, CA, United States) software were used for statistical analyses. Normally distributed measurement data were expressed as mean ± SD and compared with Student t-test, while non-normally distributed measurement data were expressed as median (interquartile range) and compared with Mann-Whitney test (non-parametric distribution). One-way analysis of variance (ANOVA) was followed by post-hoc analysis by Tukey’s test for multiple comparisons. Two-way repeated-measures ANOVA was followed by post-hoc analysis by LSD test for multiple comparisons of repeated measurement data. Survival rates were calculated using the Kaplan–Meier method and comparisons were performed using the Log-rank test. *p* < 0.05 was considered as statistically significant.

## Results

### High Expression of CCL18 in UCs

**FIGURE 1:**
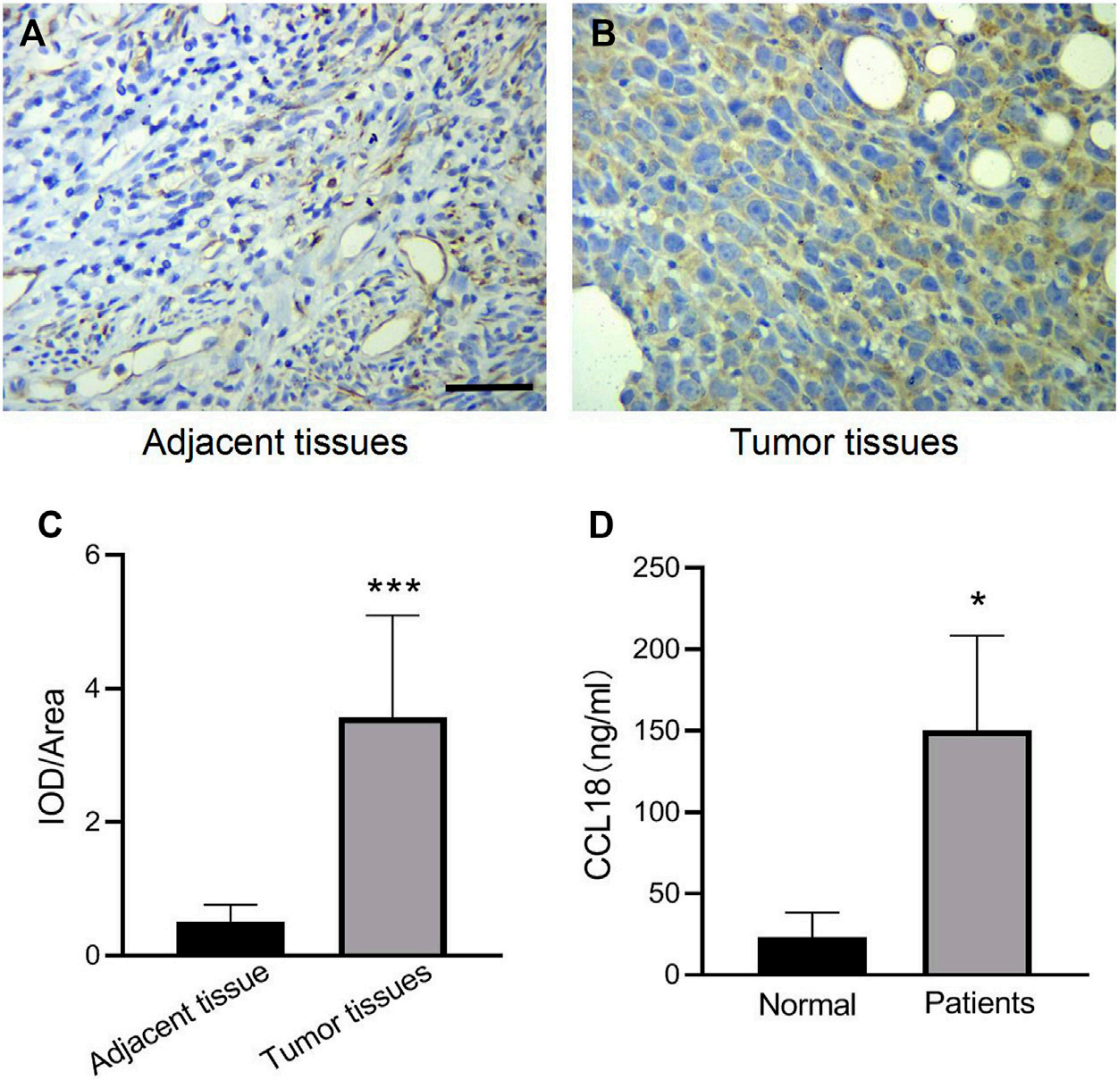
The expression of CCL18 in UCs patients was higher than that in normal subjects. **(A,B)** is the result of CCL18 immunohistochemistry in UCs tissues and adjacent cancers (400 x). **(C)** is the statistical result of the optical density of immunohistochemistry. **(D)** is the expression of CCL18 in the serum of UCs patients and healthy people. *p < 0.05 vs. Normal people, ***p < 0.001 vs. Adjacent tissue. Scale bars: 25 μm.

We first investigated the expression of CCL18 in UCs. The expression of CCL18 in UCs was significantly higher than that in normal tissues (*p* < 0.05), as shown in Figures 1A–C. Besides, compared with healthy volunteers, the serum level of CCL18 was significantly increased in patients with UCs (*p* < 0.05), (Figure 1D), indicating that CCL18 is closely related to UCs. Baseline characteristics are shown in Table 2.

**TABLE 2:** Baseline characteristics.

Characteristic	Normal (*n* = 20)	Patient (*n* = 20)
Age—yr		
Median	66	67
Range	28–87	27–83
Weight—kg		
Median	71.5	70.6
Range	65.4–75.8	63.2–74.1
Male sex—no. (%)	15/20 (75.0)	14/20 (70.0)
Current or former smoker—no./total no. (%)	12/20 (60)	13/20 (65)
Visceral disease — no./total no. (%)	11/20 (55)	12/20 (60)

### CCL18 Promotes Proliferation, Invasion, and Migration of UCs

**FIGURE 2:**
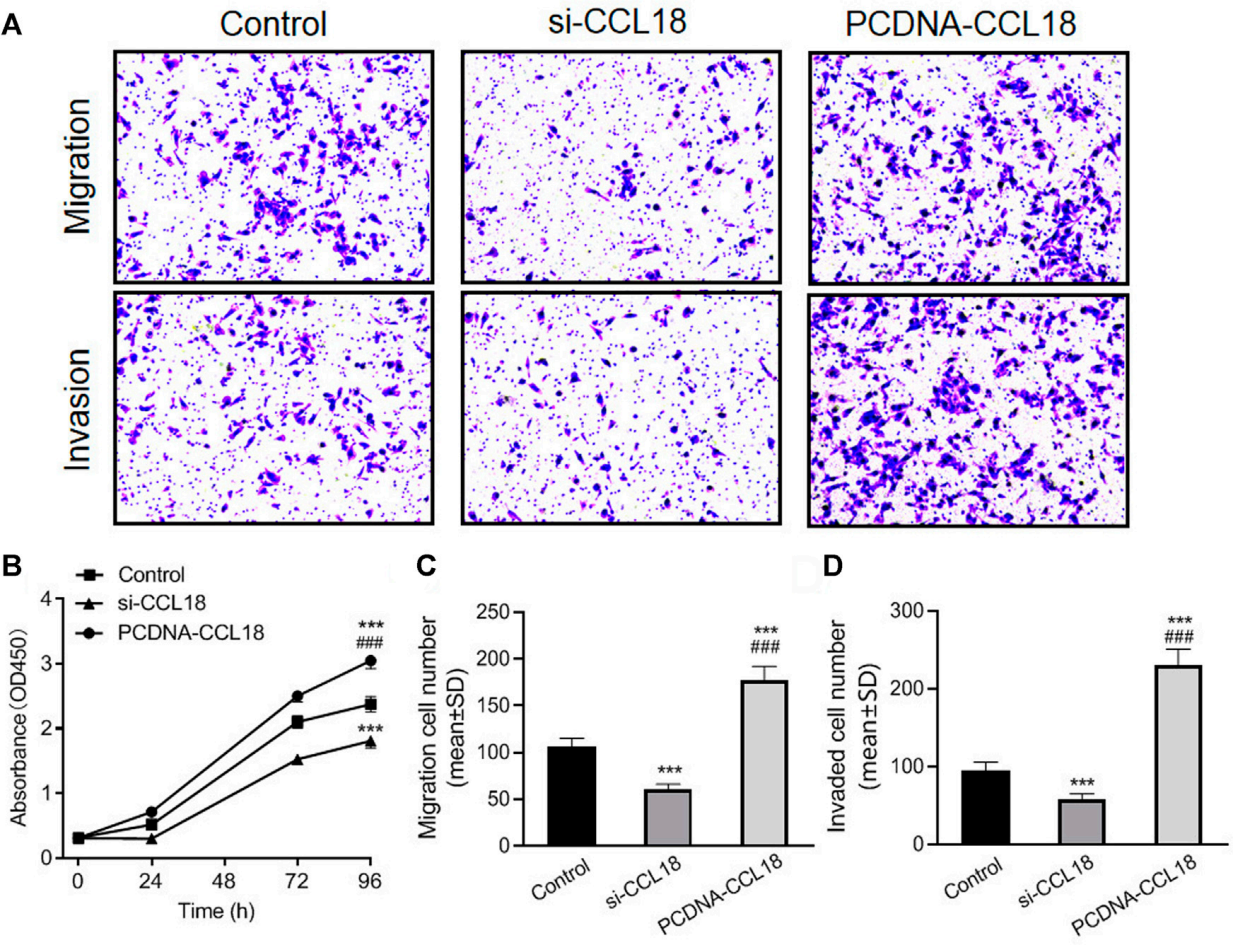
CCL18 promotes proliferation, migration, and invasion of BUC T24 cells. **(A)** is picture of Transwell cell migration and invasion assay (stained with crystal violet). The migration and invasion ability of BUC T24 was significantly enhanced after CCL18 overexpression and suppressed after CCL18 silencing. **(B)** is the statistical result of cell proliferation ability detected by CCK-8 assay. The proliferative capacity of BUC T24 was promoted by CCL18 overexpression and restricted by CCL18 silencing at 96 h time point. **(C,D)** are the statistical results of migration and invasion, respectively. ***p < 0.001 vs. Control, ###*p* < 0.001 vs. Si-CCL18.

To further confirm the effect of CCL18 on UCs, we manipulated CCL18 expression in BUC T24 cells. The proliferation of BUC T24 cells with CCL18 overexpression was significantly enhanced compared with untreated cells (*p* < 0.05). In contrast, the proliferation of cells with downregulated expression of CCL18 was inhibited (*p* < 0.05), as shown in Figure 2. In addition, CCL18 overexpression also significantly promoted the migration and invasion of BUC T24 cells (*p* < 0.05), and the opposite effect of CCL18 downregulation was observed, as shown in Figure 2.

The results of wound healing assays also showed that the wound healing degree of the CCL18 overexpression group was significantly higher than that of the untreated group and the CCL18 knockdown group (*p* < 0.05), after 12 h (Si.1). After 24 h, all the scratches were healed just in the CCL18 overexpression group.

### Prediction and Verification of miRNA Targeting CCL18

Previous studies have confirmed that miRNAs can regulate the biological processes of a variety of tumors, and CCL18 may also function as a target protein of miRNAs (Song et al., 2018; Korbecki et al., 2020). To confirm whether CCL18 was regulated by miRNAs in UCs, we screened for possible types of miRNAs. The miRanda detected 37 conservative miRNAs targeting human CCL8 mRNA, further screening out eight miRNAs with mirSVR score ≤ -0.1 and Phastcons score ≥0 (Table 3).

**TABLE 3:** mirSVR score and PhastCon score.

miRNA	mirSVR score	Phastcon score
*miR-183*	−0.9208	0.5013
*miR-374a*	−0.9214	0.5265
*miR-374b*	−0.9157	0.5272
*miR-33a*	−0.8308	0.5084
*miR-33b*	−0.8289	0.5084
*miR-128*	−0.5164	0.5486
*miR-138*	−0.2031	0.5399
*miR-410*	−0.1158	0.5271

**FIGURE 3:**
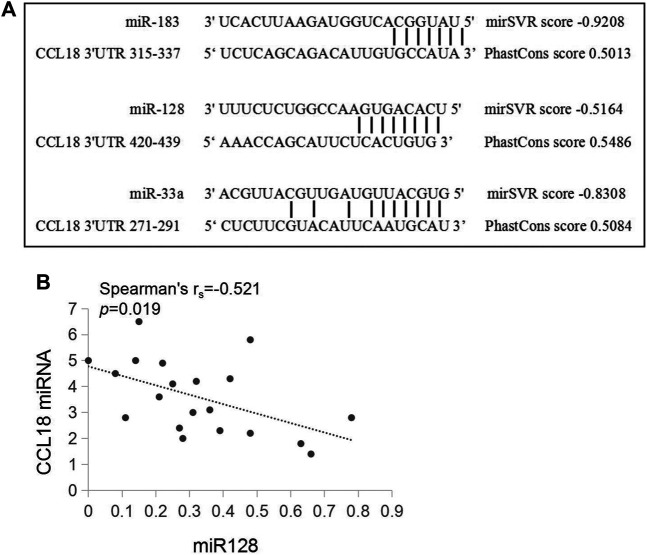
There is a pairing relationship between ccl18 and the three types of miRNAs and a negative correlation with the amount of miR128 expressed. **(A)** is a schematic of CCL18-3′UTR and miR-183, miR-128, and miR-33a sequence binding. **(B)** is the relationship between CCL18 and the relative expression amount of miR-128.

A total of 30 conservative miRNAs targeting human CCL18 mRNA were detected by total context++ score from the Targetscan. Combined mirSVR score, Phastcons score, and total context++ score, three miRNAs with high targeting, including miR-183, miR-128, and miR-33a, were screened out. The interaction between miRNAs and CCL18-3′UTR was shown in Figure 3. Furthermore, the contents of CCL18, *miR-183, miR-128 and miR-33a* in urothelial carcinoma tissues were detected, indicating a negative correlation between the content of CCL18 and *miR-128* (r_s_ = -0.521, *p* < 0.05), as shown in Figure 3. No correlation between CCL18 mRNA expression and miR-183 and miR-33a was detected (*p* > 0.05).

### HMSC-128-EV Inhibits the CCL18 Secretion of UCs

Mesenchymal stem cells (MSCs) can migrate to tumor sites and perform complex functions during tumor progression. Exosome vectors were prepared by HMSC, and exosome miRNAs were transferred into tumor cells, which have been widely used in cancer research. To further confirm the regulatory effect of *miR-128* on CCL18, we chose exosomes as the carrier of *miR-128*. Firstly, HMSC-EV was prepared and identified by CD9 and CD63 surface-specific markers of exosome (Si.3 A-C). The TEM showed that the HMSC-EVs were spherical and homogeneous in shape and size, with a diameter of about 100 nm (Si.3 D-E). Besides, the diameter and Zeta potential of the HMSC-EVs were 105.4 ± 11.4 nm and −23.7 ± 1.2 mv, respectively, indicating the surface of exosomes is negatively charged (Si.3 F). Further, HMSCs were infected with adenovirus to overexpress the *miR-128.* Then the HMSC-128-EV was produced. The expression levels of *miR-128* in the above two exosomes were detected by RT-PCR, demonstrating that *miR-128* was highly expressed in HMSC-128-EV but not expressed in HMSC-EV (Si.3 G).

**FIGURE 4:**
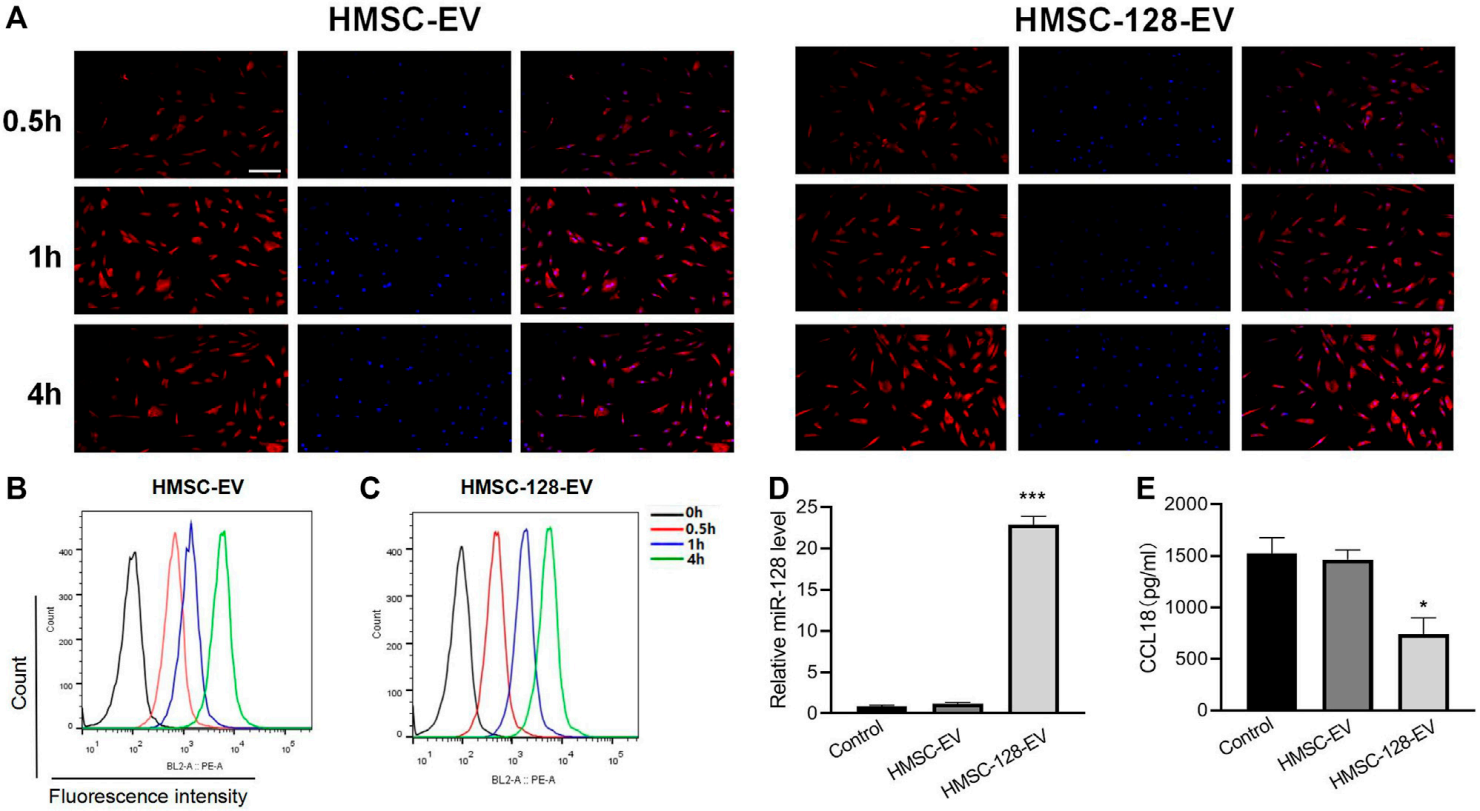
HMSC-128-EV is able to significantly increase miR-128 within BUC T24 while reducing CCL18 expression. **(A–C)** are the results of endocytosis of exosomes detected by confocal microscopy and flow cytometry, and both HMSC-EV and HMSC-128-EV could be endocytosed by BUC T24. **(D)** is the level of miR-128 within BUC T24 of each group as determined by RT-PCR, and HMSC-128-EV was able to significantly increase intracellular miR-128. **(E)** is CCL18 within BUC T24 of each group determined by ELISA, and HMSC-128-EV was able to inhibit CCL18 expression within the cells. *p < 0.05, ***p < 0.001 vs. Control. Scale bars: 30 μm.

We used HMSC-EV and HMSC-128-EV to co-culture with BUC T24 and found that exosomes could be effectively endocytosed by BUC T24 (Figures 4A–C). Meanwhile, HMSC-128-EV could significantly increase the level of *miR-128* in BUC T24 (*p* < 0.001) (Figure 4D).

To confirm the effect of *miR-128* on the synthesis of CCL18 by BUC T24, we treated BUC T24 with HMSC-DC, HMSC-128-EV. By ELISA, we found that compared with the control group, the content of CCL18 released by BUC T24 in HMSC-Ev group was not different (*p* > 0.05), while the content of CCL18 released in HMSC-128-EV group was significantly decreased (*p* < 0.05) (Figure 4E). It suggested that overexpression of *miR-128* could inhibit the synthesis and secretion of CCL18 by UCs.

### The *miR-128* can Inhibit Cell Proliferation, Migration and Invasion in UCs, Which can Be Reversed by CCL18

**FIGURE 5:**
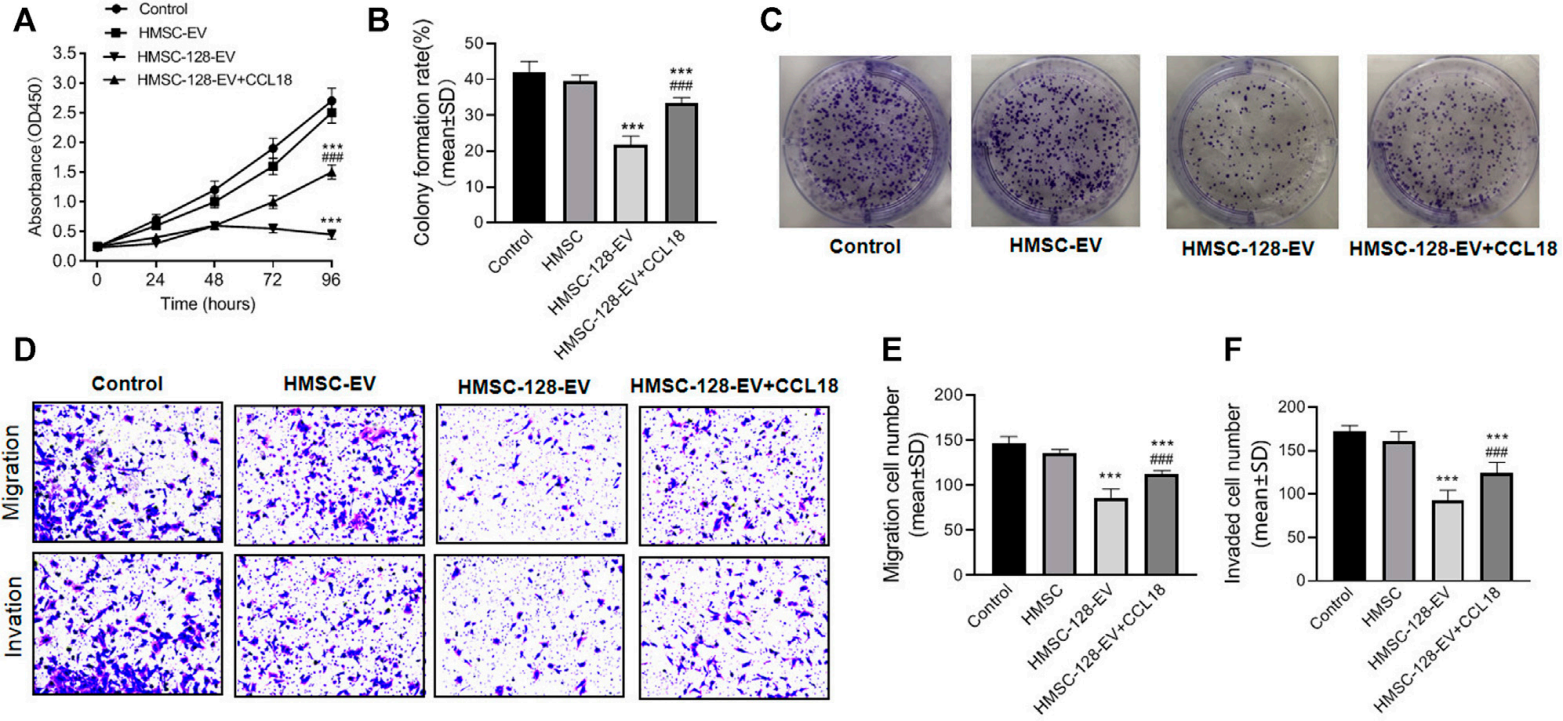
HMSC-128-EV inhibited BUC T24 proliferation, migration, invasion. **(A)** is the result of cell viability assays by CCK8, which showed that HMSC-128-EV could significantly inhibit the viability of BUC T24 when co-cultured for 96 h. **(B, C)** are the results of clonogenic assay testing cell proliferation, and HMSC-128-EV could significantly inhibit the proliferation capacity of BUC T24. **(D–F)** are the results of Transwell cell migration and invasion assays, and HMSC-128-EV could significantly inhibit the migration and invasion abilities of BUC T24. ***p < 0.001 vs. Control, ###*p* < 0.001 vs. HMSC-128-EV.

Considering that HMSC-128-EV were able to affect CCL18 expression of BUC T24, we further investigated the effect of HMSC-128-EV on the tumor process. CCK8 results showed that the cell viability of HMSC-EV groups were not different from the control group (*p* > 0.05), but it was significantly reduced in the HMSC-128-EV group (*p* < 0.001) (Figure 5A). Similarly, the numbers of clone formation in HMSC-Ev group was not different from the control group (*p* > 0.05), and was significantly reduced in HMSC-128-EV group (*p* < 0.001) (Figures 5B,C).

There was no significant differencein tumor cell migration and invasion ability between HMSC-EV group and control group (*p* > 0.05), while those in HMSC-128-EV group was significantly reduced than those in the control group (*p* < 0.001) (Figures 5D–F).

To explore the mechanisms underlying the effects of *miR-128* on the proliferation, invasion, and migration functions of UCs, we used CCL18 (0.5 ng/ml) and HMSC-128-EV to coculture with UCs. We found a significant recovery in the proliferation, invasion, and migration functions of the UCs compared with the HMSC-128-EV group (*p* < 0.001).

### The *miR-128* can Promote Apoptosis of UCs Cells, Which can be Reversed by CCL18

**FIGURE 6:**
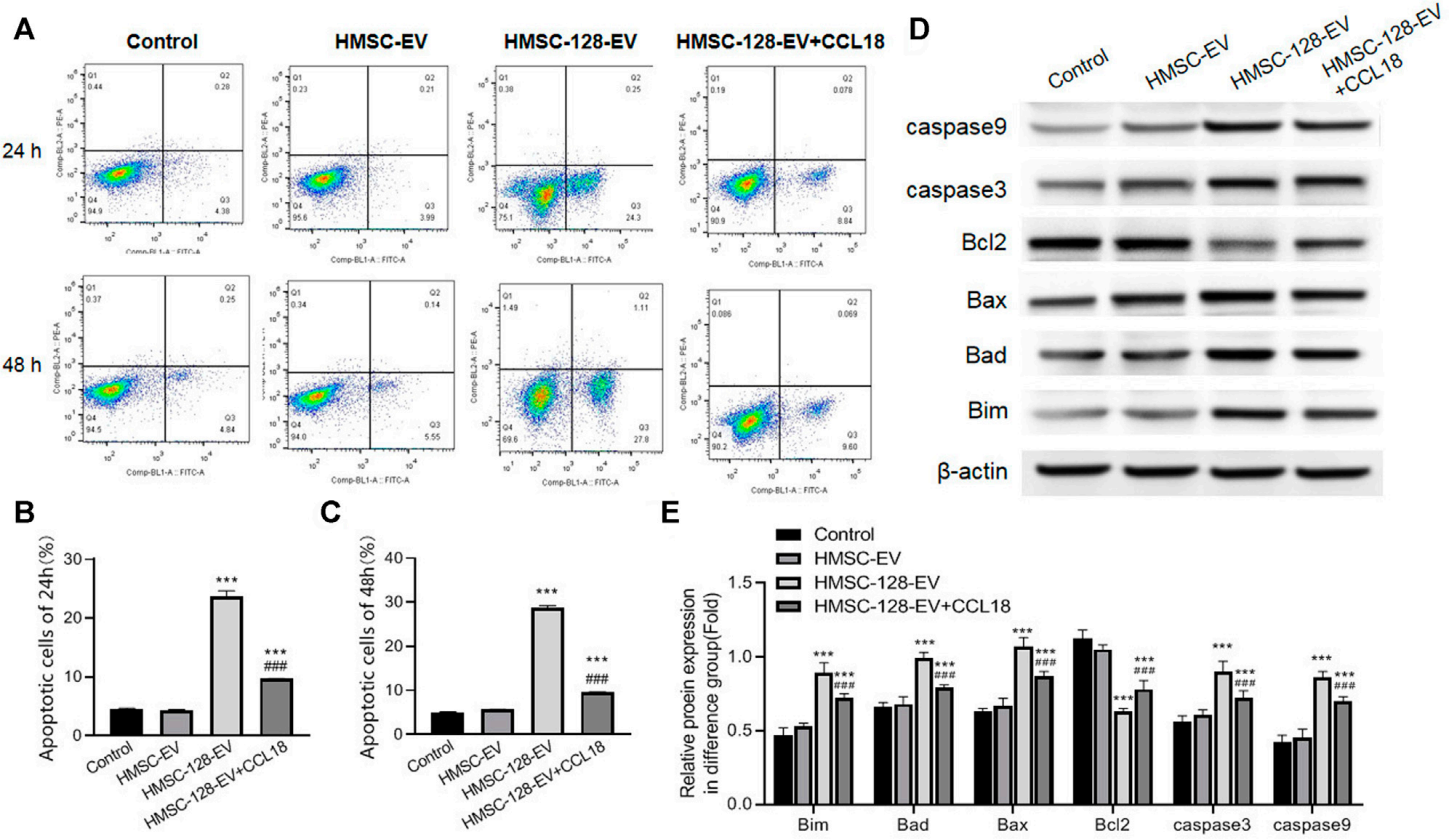
HMSC-128-EV promoted BUC T24 apoptosis. **(A)** is the result of apoptosis detection using flow cytometry (stained by annexin/PI). **(B, C)** are the apoptosis rates after co-culture for 24 and 48 h. **(D, E)** are the western blotting typical picture and statistical results of Bim, Bad, Bax, caspase 3, caspase 9 and Bcl2 in each group, respectively. HMSC-128-EV was able to promote the expression of BUC T24 apoptotic protein and inhibit the expression of survival factors. ***p < 0.001 vs. Control, ###*p* < 0.001 vs. HMSC-128-EV.

Besides, we also explored the apoptosis situation of tumor cells. The apoptosis rates of tumor cells in HMSC-EV groups was not different from the control group (*p* > 0.05), while the apoptosis rate in the HMSC-128-EV group was significantly higher than that of the control group (*p* < 0.001) (Figures 6A–C).

In terms of a series of protein markers associated with tumor cells apoptosis and survival, on the one hand, the pro-apoptotic proteins including Bim, Bad, Bax, caspase 3, and caspase 9 in each group were also detected, indicating that compared with the control group, the protein expressions of HMSC-128-EV group were significantly higher (*p* < 0.05) (Figures 6D,E). On the other hand, the level of cell survival-promoting factor Bcl2 was the lowest in the HMSC-128-EV group (*p* < 0.001). Taken together, *miR-128* silencing CCL18 may promote apoptosis of UCs cells.

In addition, we found that exogenous supplementation with CCL18 (0.5 ng/ml) resulted in a significant reduction (*p* < 0.001) in the proportion of cells undergoing apoptosis and in the detection of apoptosis related proteins (Bim, Bad, Bax, caspase 3, and caspase 9) compared with the HMSC-128-EV group. The cell survival promoting factor Bcl2 was significantly elevated (*p* < 0.001).

### The *miR-128* can Inhibit Epithelial-Mesenchymal Transition of UCs, Which can Be Reversed by CCL18

**FIGURE 7:**
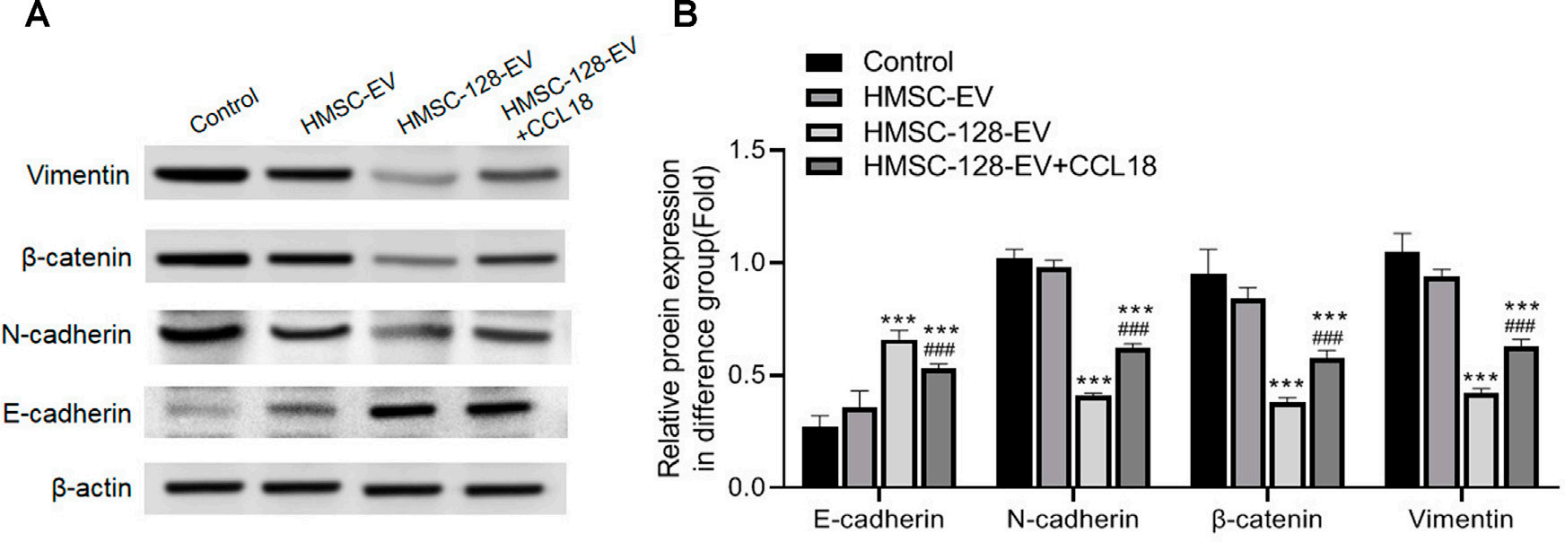
HMSC-128-EV inhibited EMT of BUC T24. **(A)** is the typical picture of western blotting of E-cadherin, N-cadherin, beta-catenin, and Vimentin. **(B)** is the statistical results of western blotting of E-cadherin, N-cadherin, Beta-catenin, and Vimentin. HMSC-128-EV significantly inhibited N-cadherin, Beta-catenin, and Vimentin expression and promoted E-cadherin expression. ***p < 0.001 vs. Control, ###*p* < 0.001 vs. HMSC-128-EV.

EMT is a major feature of UCs, we further explored on the effect of HMSC-128-EV on the EMT of UCs. Compared with the control group, after treatment of *miR-128*, the intracellular E-cadherin expression was significantly increased (*p* < 0.001), and the expression of N-cadherin, β-catenin, and Vimentin was significantly decreased (*p* < 0.001) (Figures 7A,B).

After exogenous supplementation with CCL18, N-cadherin, β-catenin, and Vimentin significantly increased (*p* < 0.001) and E-cadherin significantly decreased (*p* < 0.001) compared with the HMSC-128-EV group. It is suggested that blocking CCL18 by *miR-128* may inhibit the EMT of UCs cells, thereby regulating tumor invasion.

### The *miR-128* can Significantly Inhibit the Growth of UCs in Nude Mice

**FIGURE 8:**
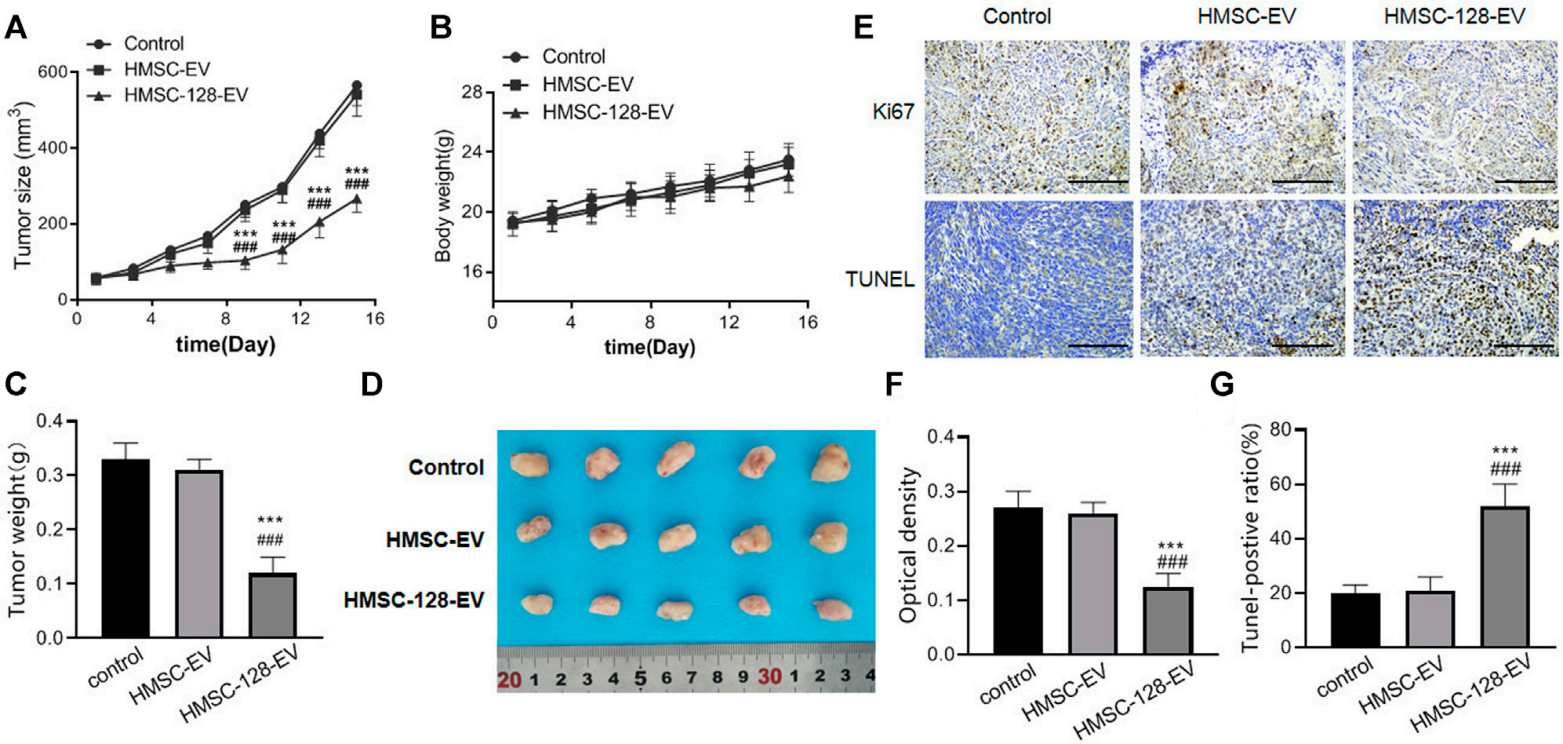
HMSC-128-EV inhibited the growth of UCs in nude mice. **(A, B)** are line charts of the tumor volume and the body weight of nude mice in each group over time, respectively. **(C)** is the weight of tumors in each group after 16 days of treatment. **(D)** is a typical diagram of tumors from each group after 16 days of treatment. **(E)** is the immunohistochemical staining results of Ki67 and TUNEL of tumor tissues in each group. **(F, G)** are the statistical results of Ki67 and TUNEL, respectively. HMSC-128-EV significantly inhibited the proliferation and promoted the apoptosis of UCs in nude mice. *p < 0.05, **p < 0.01, ***p < 0.001 vs. Control. #*p* < 0.05, ##*p* < 0.01, ###*p* < 0.001 vs. HMSC-EV. Scale bars: 100 μm.

To confirm the therapeutic effect of HMSC-128-EV *in vivo*, we injected HMSC-128-EV into the tail vein of nude mice transplanted with UCs. For monitoring the growth of xenograft tumors, tumor volume and weight were measured in all mice. The results showed that the tumor volume and weight of mice treated with HMSC-128-EV decreased significantly, compared with control and HMSC-EV groups (*p* < 0.05) (Figures 8A,C,D). Meanwhile, during the administration, the weight of mice increased steadily, and no weight loss and other adverse symptoms were observed (Figure 8B). Furthermore, in order to reflect the status of proliferation and apoptosis, Ki67 and TUNEL staining were performed on the transplanted tumor cell. Compared with the control and HMSC-EV groups, the mean optical density value of Ki67 positive was significantly reduced in the HMSC-128-EV group (*p* < 0.001), while the proportion of Tunel-positive tumor cells was significantly increased (*p* < 0.001) (Figures 8E–G).

### The *miR-128* can Inhibit the Metastasis of UCs and Extend the Lifespan of Nude Mice

**FIGURE 9:**
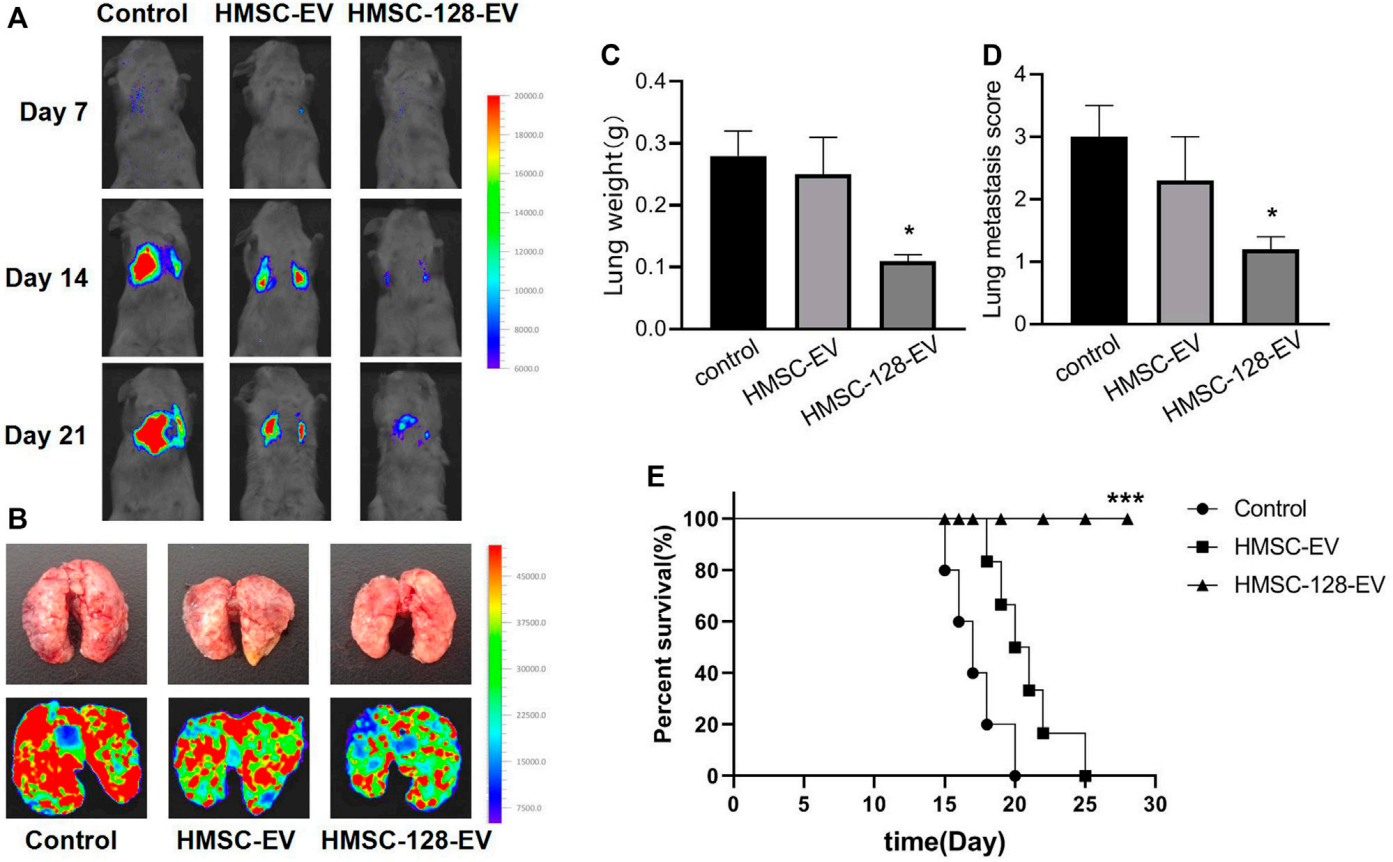
HMSC-128-EV inhibited the metastasis of UCs and prolonged the survival time of nude mice. **(A,B)** typical images of live nude mice and lung fluorescence imaging, respectively. **(C,D)** are the statistical results of lung tissue weight and the number of lung metastatic nodules in nude mice of each group, respectively. HMSC-128-EV could significantly reduce the lung metastasis of UCs in nude mice. **(E)** is the survival curve of nude mice in each group. HMSC-128-EV can significantly prolong the survival time of nude mice with UCs. *p < 0.05, ***p < 0.001 vs. Control.

To evaluate the inhibitory effect of HMSC-128-EV on the metastasis of UCs, we monitored lung metastases in nude mice. For the control and HMSC-EV groups, the fluorescence intensities in the lungs of mice increased gradually with time (Figure 9A). On the 21st day, compared with the control and HMSC-EV groups, the fluorescence brightness, the weight of lung tissue, and the number of metastatic nodules in mice of the HMSC-128-EV group were significantly decreased (*p* < 0.05) (Figures 9B–D). Moreover, the survival rate of mice in the HMSC-128-EV group was significantly increased (*p* < 0.05) (Figure 9E).

## Discussion

In this study, we found that CCL18 was highly expressed in UCs, and down-regulation of CCL18 can inhibit proliferation, migration, and invasion of UCs. CCL18 may be regulated by *miR-128*. Exosome delivered *miR-128* can significantly inhibit CCL18 synthesis of UCs cells. DNA sequence alignment also detected a binding site between CCL18 and *miR-128*, and the expression of the two was negatively correlated in UCs cells. Further studies found that *miR-128* significantly inhibited the proliferation, migration, and invasion, and promoted apoptosis of UCs cells. On the animal model, we also confirmed that *miR-128* can inhibit the proliferation and metastasis of UCs in nude mice, and prolong their survival time. Our findings shed new light on the therapeutic innovation of UCs. The *miR-128*, loaded in exosomes, has the potential to become a new therapeutic agent for UCs.

CCL18 is a kind of chemokine, predominantly expressed by monocyte-derived cells with M2 phenotype and dendritic cell (Kodelja et al., 1998; Schutyser et al., 2005). CCL18 is not only a constitutive product under normal conditions but also an inducible chemokine under inflammatory conditions (Schutyser et al., 2005). Previous studies have demonstrated a strong correlation between CCL18 expression and various malignancies, such as ovarian cancer, gastric cancer (Schutyser et al., 2002; Leung et al., 2004). Our study found that CCL18 was highly expressed in the UCs. This finding is the same as previous studies (Liu X. et al., 2019). Inhibition of CCL18 expression can significantly inhibit the proliferation, invasion, and migration of BUC T24, indicating that the high expression of CCL18 plays an important role in the maintenance of UCs tumor process. Therefore, artificially regulating CCL18 expression may have therapeutic effects on UCs.

Many studies have proved that miRNA can regulate the growth, differentiation, and apoptosis of tumor cells (Tong and Nemunaitis, 2008; Shimono et al., 2009). Altered expression of multiple miRNAs including *miR-21, miR-155* was associated with tumorigenesis (Zhang et al., 2008; Mattiske et al., 2012). In addition, some miRNAs, such as let-7, *miR-34a,* et al. possess tumor suppressor functions (Akao et al., 2006; Liu et al., 2011). The *miR-128* has been demonstrated to play an important role in the occurrence, development, and targeted therapy of colorectal, prostate, and ovarian cancers (Khan et al., 2010; Li et al., 2014; Liu T. et al., 2019). For the UCs, multiple miRNAs have been confirmed to participate in the biological process (Song et al., 2010; Fujii et al., 2015). But the role of *miR-128* in the tumor process of UCs has not been reported. In this study, we found that *miR-128* played an important role in the proliferation and metastasis of UCs. In this study, we found that *miR-128* was functionally linked to the secretion of CCL18 of UCs. It was able to significantly inhibit CCL18 synthesis of BUC T24. Meanwhile, *miR-128* loaded in exosomes was able to obviously inhibit cell proliferation, metastasis, and invasion of UCs. This suggests that *miR-128* may regulate the tumorigenic process of UCs by regulating CCL18 secretion from BUC T24 in the tumor microenvironment. This may be the mechanism by which miR-128 has therapeutic potential for UCs.

By bioinformatics analysis, we found that CCL18 was a target protein of *miR-128*. The *miR-128*, loaded in exosome, was able to inhibit CCL18 secretion from BUC T24. Our study also found that the effect of *miR-128* on the proliferation, invasion and migration of UCs can be reversed by CCL18. In addition, we also found that *miR-128* promoted apoptosis and inhibited EMT of UCs could be reversed by CCL18. These indicated that the effect of *miR-128* on the biological process of UCs was directly related to CCL18. This pathway was first identified in UCs and enriched the regulatory mechanism of the tumor microenvironment of UCs, and its significance as an avenue for targeted therapy of UCs warranted further investigation.

In addition, our innovative use of embryonic stem cell exosomes as a delivery vehicle for *miR-128* confirmed its ability to efficiently elevate *miR-128* levels in BUC T24, and affected its function. Exosomes are saucer-shaped vesicles released by cells to carry membrane and cytosolic components, with a diameter of 30–100 nm (Simons and Raposo, 2009). Exosome could bear combinations of ligands, bind to target-cell membranes, and fuse with target cells (Théry et al., 2002). Thus, exosomes can be used as drug delivery vehicles and have a good delivery effect (Batrakova and Kim, 2015; Haney et al., 2015). In this study, we confirmed that exosomes not only could deliver *miR-128* to BUC T24 but also had a promising therapeutic effect in animal experiments. These findings further support the possibility that *miR-128* in combination with exosomes could be used to treat the UCs. This innovation in miRNA delivery modality was the first reported in UCs. It provided a new way for the application of miRNAs in the treatment of UCs.

The present study also has limitations. UCs are capable of secreting a variety of cytokines in the tumor microenvironment, we only explored the role of CCL18 and did not investigate whether other cytokines also contribute to UCs. We will further refine it in the future studies.

## Conclusion

Our study innovatively found that *miR-128* could influence the tumor process of the UCs by regulating CCL18 secretion. Exosomes have the potential to function as a carrier of *miR-128* in the treatment of UCs.

## Data Availability

The original contributions presented in the study are included in the article/supplementary material, further inquiries can be directed to the corresponding author.
